# Fluid flow behaviour in vesicular basalt samples from the Skoll High, Vøring Margin

**DOI:** 10.1007/s40948-025-01080-9

**Published:** 2025-12-18

**Authors:** Peter Betlem, Marija Plahter Rosenqvist, John Millett, Luke Griffiths, Irina Filina, Joonsang Park, Sverre Planke, Kim Senger, Elin Skurtveit

**Affiliations:** 1 Norwegian Geotechnical Institute, Ullevaal Stadion, P.O. Box 3930, 0806 Oslo, Norway; 2 Department of Arctic Geology, The University Centre in Svalbard, P.O. Box 156, 9171 Longyearbyen, Norway; 3 Department of Geosciences, University of Oslo, P.O. Box 1047, 0316 Oslo, Norway; 4 The Njord Centre, University of Oslo, P.O. Box 1048, 0316 Oslo, Norway; 5 Volcanic Basin Energy Research, Blindernveien 5, 0361 Oslo, Norway; 6 Department of Geology and Geophysics, University of Aberdeen, King′s College, Aberdeen, AB24 3FX, UK; 7 Department of Earth and Atmospheric Science, University of Nebraska-Lincoln, P.O. Box 880340, Lincoln, NE 68588-0340 USA

**Keywords:** International Ocean Discovery Program (IODP) Expedition 396, Carbon sequestration, Mid-Norwegian continental margin, Lava flows, Fluid flow experiments, Clay swelling

## Abstract

This study presents the fluid flow testing results of 32 lava flow samples recovered from IODP Sites U1571/U1572 (145–315 mbsf) on the Skoll High, Vøring Margin. Few past studies have investigated the mid-Norwegian margin’s basalt reservoir and flow properties. Even fewer implement subsurface-like conditions to explore in-situ fluid flow and the possible response to changing pore fluids during injection. These aspects are critical for large-scale carbon sequestration. In this study, hydraulic properties including porosity and permeability were measured at ambient conditions for all samples along with multi-stress, multi-fluid core flooding experiments on a representative sample enclosed in an X-ray µCT scanner. Petrographic observations reveal varying degrees of alteration with secondary clays, including smectite, commonly lining pores and vesicles. He-porosities (4.2–45.6%) fall mostly below the theoretical percolation threshold of ~ 30% for randomly distributed spherical pores. Klinkenberg-corrected nitrogen-gas permeability (0.0038–361.9 mD) shows weak correlation to porosity and no apparent depth dependence. Further, brine permeability is four orders of magnitude lower than gas permeability, with no notable flow reduction during injection of CO_2_. Rather than being stress-dependent, we argue that brine-induced swelling significantly reduced the accessibility of microstructural elements to fluids. The findings urge caution when using literature-reported, ambient gas measurements for basalts with similar levels of alteration as they may not accurately reflect in-situ fluid flow properties. Low matrix permeability of the moderately to highly altered vesicular basalts and prevalence of swelling clays may, without major contributions from fractures, limit the injectivity potential into the basaltic lava flows on the Skoll High, Vøring Margin.

## Article highlights

- Core flooding experiments indicate brine permeabilities roughly 4 orders of magnitude lower than to nitrogen gas.
- Fines and clay interactions narrow microstructural elements and render the pore space inaccessible to fluids.
- Low matrix permeability and prevalence of swelling clays limits injection potential in the tested basalts.

## Introduction

Sub-aerial lava flow hosted reservoirs are utilized globally for various sub-surface applications ranging from water aquifers, hydrocarbon reservoirs, geothermal energy and more recently, CO_2_ storage (Millett et al. 2024). Following the technical successes of the CarbFix and Wallula projects (McGrail et al. 2017; Ratouis et al. 2022; Snæbjörnsdóttir et al. 2020), the exploration and appraisal of basalt carbon sequestration targets is expanding globally (Millett et al. 2024; Snæbjörnsdóttir et al. 2020), with renewed interest for both onshore and offshore settings (Jackson et al. 2021; Millett et al. 2024; Planke et al. 2023d; Rosenqvist et al. 2024). Yet, large-scale implementation requires further research and development (Matter et al. 2011), particularly focusing on the derisking of hydraulic properties such as effective porosity and permeability (Zakharova et al. 2012).

The overall hydraulic properties and injectivity of basalt lava flows can be highly sensitive to physiochemical and mechanical changes, especially when they comprise poorly or largely unconnected vesicular pore networks (Behnsen and Faulkner 2012; Davy et al. 2007; Faulkner and Rutter 2000; Luquot and Gouze 2009; Noiriel et al. 2004; Tanikawa and Shimamoto 2009). Here, burial depth, loading and unloading, alterations, age, temperature, physiochemical interactions and other mechanical responses may have a particularly outsized role in determining flow and chemical reactivities (Cant et al. 2018; Heap and Violay 2021; Lamur et al. 2017; Marieni et al. 2021; Marins et al. 2022; Millett et al. 2024; Pollyea et al. 2014; Rosenqvist et al. 2024, 2023). While the typical distribution of permeable zones within lava flow sequences is relatively well known, i.e., typically in the lava flow tops (Millett et al. 2024), uncertainty remains as to the three-dimensional distribution and connectivity of these zones across larger areas. Both vary significantly depending on a range of magmatic, emplacement, and environmental variables (Marins et al. 2022; Navarro et al. 2020; Pollyea et al. 2014). Notably, the evolution and extent of such zones are intrinsically linked to their internal structures, including pores and microcracks, pore geometry and connectivity, alteration state of basalts, and the presence of fines and clays (Heap et al. 2018; Millett et al. 2024; Navarro et al. 2020; Rosenqvist et al. 2023).

The presence of secondary minerals and clays plays a particularly important role. Swelling clay minerals such as smectite are frequently observed in basalts and are usually one of the earliest alteration minerals formed from water–rock interactions at low-temperature conditions (Neuhoff et al. 1999; Seyfried and Bischoff 1979). Smectites may line vesicles and microfractures, narrowing pore throats and reducing effective connectivity (Aksu et al. 2015; Barreto et al. 2017; Callow et al. 2018). Clay behaviour is highly sensitive to fluid composition and stress state: swelling, remobilization, and fines migration can all drastically lower permeability (Behnsen and Faulkner 2012; Callow et al. 2018; Heap et al. 2018). Consequently, the occurrence and properties of clays represent a first-order control on reservoir quality in altered basalt sequences, making their role central to evaluating injectivity and storage capacity in volcanic CO_2_ storage targets. Crucially, there remain discrepancies between findings in the laboratory and those under in situ conditions, especially where it concerns fluid flow properties such as permeability and porosity (Heap et al. 2018, and references therein).

To complement seismic, site and well-scale evaluations, fit-for-purpose and advanced laboratory tests on core samples are critical for characterizing site-specific reservoir and fluid flow properties. The fluid flow potential of basalt samples is typically appraised through effective porosity and Klinkenberg-corrected gas permeability measurements at ambient or near-ambient pressures in the lab (Heap et al. 2018; Millett et al. 2024). While many experimental studies use an inert gas as the pore fluid, only some include water or brine (e.g., Gaunt et al. 2014; Kendrick et al. 2013; Kolzenburg et al. 2012). Fewer even investigate the impact of fluids and fluid-fines interactions on effective permeability (Callow et al. 2018; Heap et al. 2018). This is perhaps surprising, given the plethora of experiments that have investigated and indicated basalt-CO_2_-water and related interactions (Lu et al. 2024; Okoli et al. 2024, and references therein). Fluid-dependent permeability differences are well known for rocks containing clays and other reactive constituents (Behnsen and Faulkner 2012; Davy et al. 2007; Faulkner and Rutter 2000; Tanikawa and Shimamoto 2009), and measurements of darcy units may vary an order of magnitude or more (Donnelly et al. 1980; Faulkner and Rutter 2000; Heap et al. 2018). This causes significant uncertainty for the appraisal of sequestration targets when only hydraulic data derived from inert gases measurements are available. Thus, measurements of in situ reservoir performance are clearly preferable to fully understand geochemical and rock-fluid interactions.

With few exceptions (Marins et al. 2022; Pollyea et al. 2014), in situ flow data are often unavailable for emerging volcanic sequestration targets, such as the mid-Norwegian Continental Margin. The margin’s Paleogene basalt sequences may be particularly suitable for the safe, permanent, and large-scale offshore sequestration of CO_2_ (Millett et al. 2024; Planke et al. 2021; Rosenqvist et al. 2023, 2023). Petroleum industry drilling and drilling by three generations of the scientific ocean drilling programs (Eldholm et al. 1989 (ODP 104); Planke et al. 2023d (IODP 396); Talwani et al. 1976 (DSDP 38)) across the Vøring Margin has provided some insight to the porosity and permeability potential of the identified lava flow sequences (Harris and Higgins 2008; Planke et al. 2023a, 2021; Planke and Eldholm 1994). At ODP Hole 642E, sustained heat flow anomalies have persisted for more than two decades and indicate relatively high bulk permeabilities (10^−13^ m^2^, ~ 10^2^ mD). Such borehole-derived estimates capture effective scale lengths on the order of kilometres to tens of kilometres (e.g., Becker and Davis 2003), extending potential recharge pathways to major structural lineaments such as the Vøring and Jan Mayen fracture zones where basement is exposed to the ocean (Harris and Higgins 2008).

**Fig. 1:**
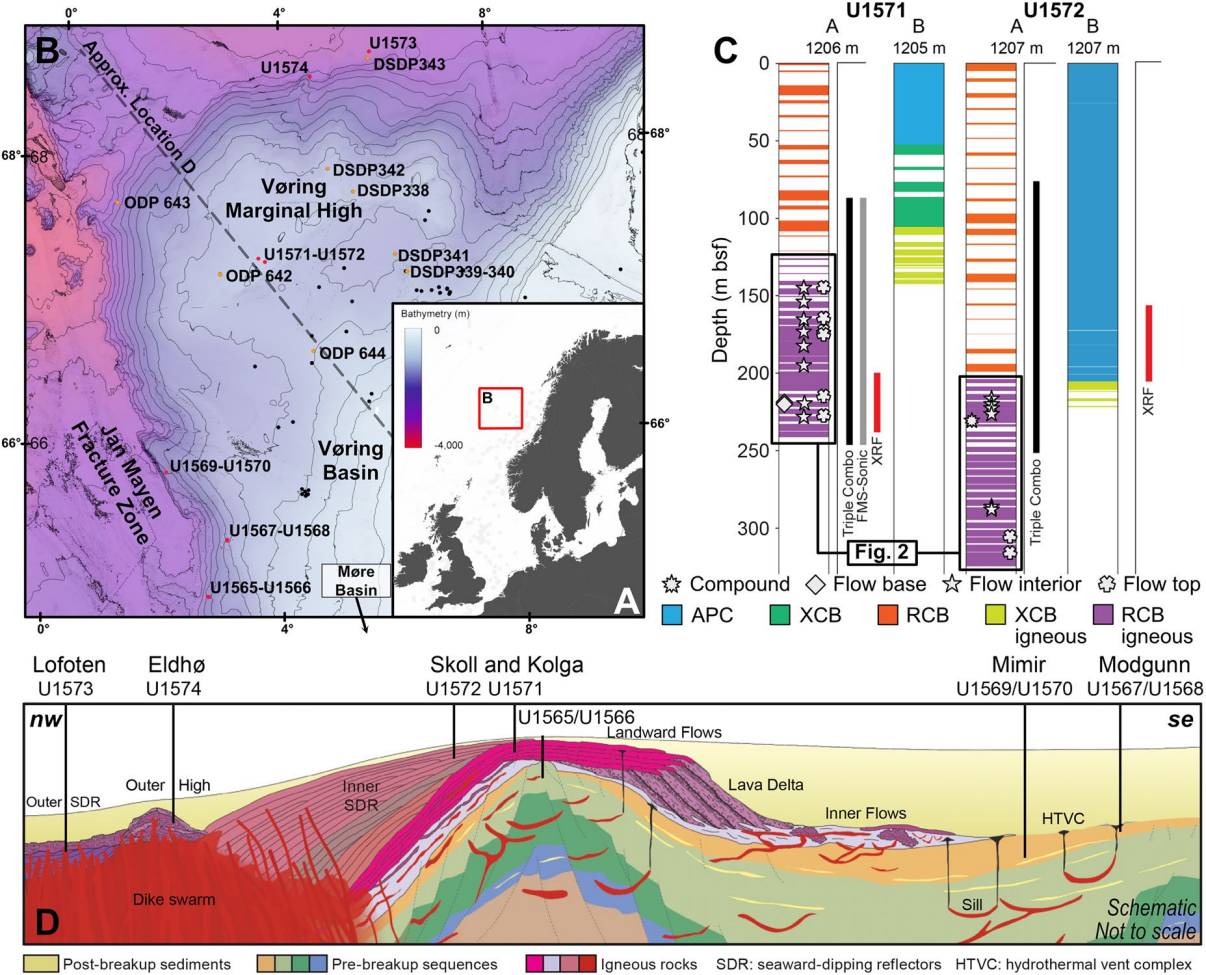
The location of the mid-Norwegian Continental Margin and area of interest explored during IODP Expedition 396 and previous activities. **A** Location of the Vøring Margin and Basin within the continental margin and offshore area marked by the red bounding box shown in B. **B** Bathymetric map and regional drilling activity. IODP sites are shown in red, while past scientific drilling activity (DSDP, ODP) and commercial boreholes are marked in orange and black, respectively. **C **Recovery, borehole method, downhole measurements, and sample type, and sampling depths for Sites U1571 and U1572. D) Schematic cross section (along dashed line in B) of the volcanic seismic facies’ unit positions, including projected positions along the margin of the sites targeted during IODP Expedition 396. Subfigures are adapted and compiled from Planke et al. (2023a). m bsf: metres below sea floor; APC: advanced piston corer; XCB: extended core barrel; RCB: rotary core barrel

While borehole observations indicate a regional reservoir potential, no dedicated fluid flow studies of the Vøring Margin basalts have been conducted so far. Filling this gap, we conducted a comprehensive testing campaign on 32 vesicular pahoehoe basalt samples recovered from IODP Sites U1571 and U1572 (Fig. 1) targeting the Skoll High lava flows on the Vøring margin. Tests included ambient pressure permeability measurements, CO_2_ and brine flow tests on a representative sample, X-ray µCT imaging, and petrographic observations. The latter was key to determining the degree of alteration and the occurrence and role of clays present across the pore space. The findings and results serve as a valuable reference to the Vøring margin’s carbon sequestration potential in basalts. They also aid ongoing efforts to understand the margin’s fluid flow behaviour across scale and time.

### Geological setting

IODP Expedition 396 drilled 21 boreholes across 10 sites on the mid-Norwegian margin (Fig. 1a). The northwest trending Jan Mayen Fracture zone segments the margin, separating the Møre and Vøring margins (Fig. 1b). Both margin segments are characterised by differing tectonomagnetic styles and sediment distributions, with the Vøring segment featuring the largest magmatic accumulation (Planke et al. 2023d). The relative position of Outer and Inner Seaward Dipping Reflectors (SDRs), Landward and Inner flows, and pre-breakup sediment sequences across the margin is shown in the schematic cross section depicted in Fig. 1d. Figure 1d also highlights the sequences targeted by the respective Expedition 396 site locations. In total, four boreholes targeted the Inner SDRs and were drilled at Sites U1571 and U1572 on the Skoll High, Vøring Margin (Planke et al. 2023b), forming the focus of this study.

Based on the extensive overview provided by (Planke et al. 2023d, 2023b), we here summarise the geological setting of the Vøring Marginal High with a focus on the Skoll High. Seismic reflection data reveal significant temporospatial variations within the Vøring Marginal High’s volcanic emplacement environments, including at the U1571 and U1572 drill sites. Site U1571 is found on the eastern part of the margin. This part is dominated by a lava flow field that exhibits incised valleys reflective of erosional features running from west to east. The field flows towards the escarpment and is offset by numerous north–south trending normal faults. Site U1572 lies further west (around 6.5 km from the U1571 site) on the pitted, western top basalt surface which features numerous anomalies and a rough morphology indicative of lava flowing across a wet substrate. The morphological differences between the sites suggest diachronous emplacement in different environments, transitioning laterally from U1572 to a potentially more distal volcaniclastic-dominated setting in U1571.

**Fig. 2:**
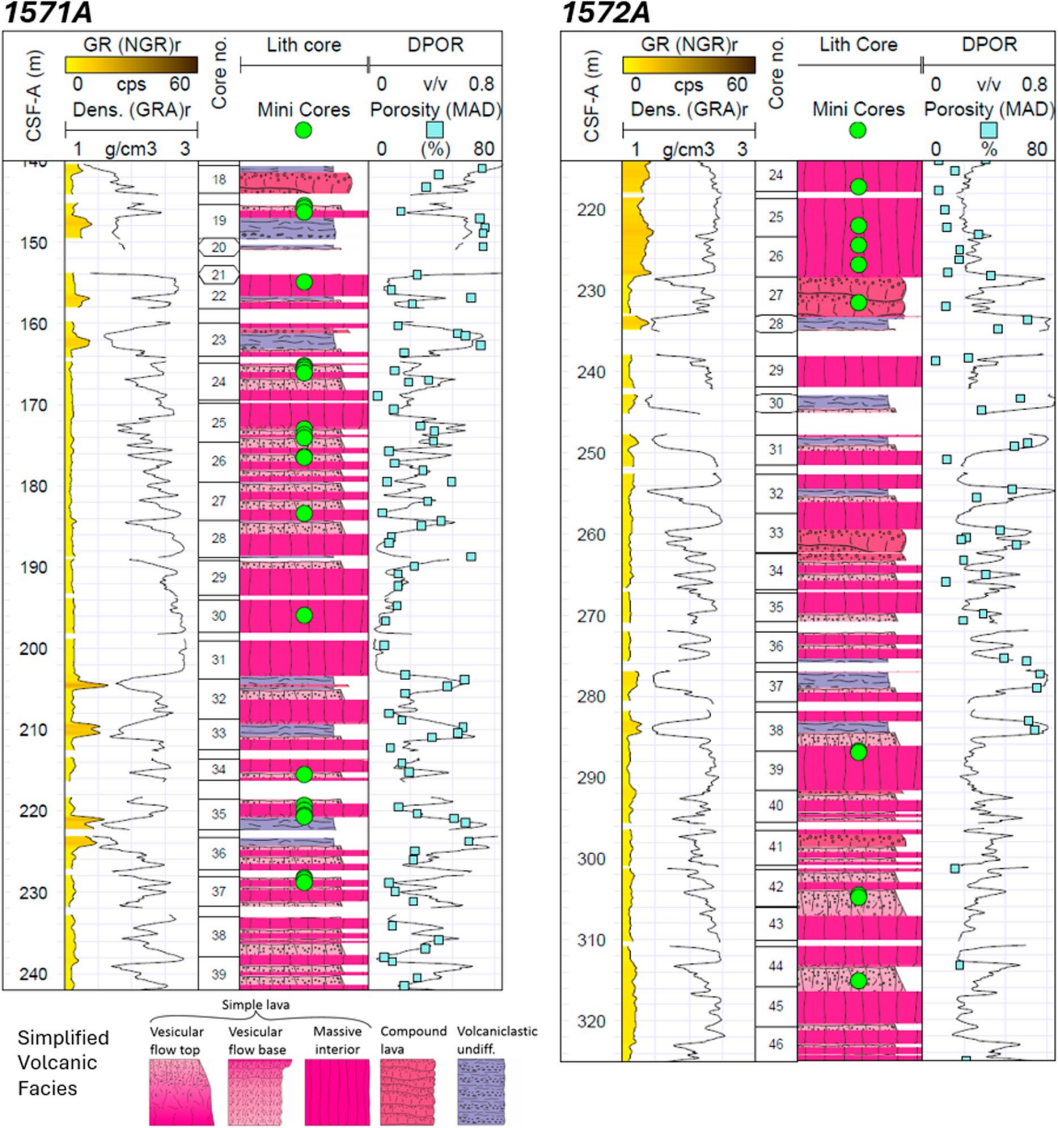
Lithostratigraphy and facies description of the U1571A and U1572A boreholes. Minicored interval depths are highlighted

The primary and first holes drilled at each site (labelled as A holes according to IODP terminology) targeted the igneous sequences, whose cored lava flow intervals were strategically sampled (Fig. 1c , 2) for the appraisal of fluid flow properties and the assessment of reservoir conditions and injection potential. In the two A holes, around 130–200 m of mud, bio-fossil oozes and hyaloclastites overlie the volcanic sequence (Fig. 1c), which comprises mainly subaerially emplaced basaltic and basaltic andesite lava flows, interbedded by thin volcaniclastic sand and mudstones. A simplified volcanic facies log for each well, based on core observations is presented in Fig. 2. The lava flows are of a pahoehoe facies and simple lava upper and lower margins and massive interiors are outlined (Millett et al. 2021). For intervals where recovered core precludes identification of clear lava flow internal facies divisions, a compound lava flow category is assigned. The lava flows often display an internal segregation in vesicle distribution with more vesicular lava flow margins (top and base) and a less vesicular interior. The highly vesicular flow tops typically feature the highest porosity and most permeable intervals in the basaltic sequences, therefore, presenting the highest reservoir potential (Rosenqvist et al. 2023). This is also evident from the wireline-derived porosity. Locally, bulbous pillow lava are present that supplement the typical lava flow facies, compound flows, and interbedded sediments.

## Data and methods

### Sample recovery and ambient analyses

#### Core recovery

Upon recovery, the cores and core plugs were treated according to IODP protocol (Planke et al. 2023c). Onboard measurements were made for all recovered core material, providing stratigraphic, geochemical and physical properties and samples that supplement downhole wireline log measurements. Following core track measurements (Planke et al. 2023c) and prior to full core splitting, core plugs were drilled horizontally from the full-diameter core with a fly-press drill onboard the JOIDES Resolution scientific drillship. Core offcuts were collected during cutting of the plugs.

#### Sample selection and description

The samples collected from U1571A and U1572A all comprise variably vesicular and variably altered basalt samples (Fig. 2). Core plug sampling primarily targeted vesicular flow margin samples, in addition to more massive lava flow core samples to cover the full range of lava flow intra-facies encountered within the wells. Samples from lava flow top and base intra-facies were typically selected based on the presence of visual porosity, thus targeting unfilled/open or partially unfilled vesicles. Sampling also avoided fractures and brecciated intervals. No samples were taken directly from the edges of core pieces. The number of samples collected was restricted due to archiving and the fact that they must be collected in the short time window prior to core splitting. In total, 32 core plugs (Table 1) were collected from the subaerial basaltic lava flows including flow top (n = 15), flow interior (n = 14), flow base (n = 2) and compound (n = 1) intra-facies from a range of depths below seafloor (145.48–315.19 m below sea floor, mbsf). Plug diameters of the cored samples range from 2.51 to 2.54 cm (mean: 2.52 cm) and lengths from 2.48 to 4.84 cm (mean: 4.43 cm).

**Table 1:** Overview of the 32 samples used in this study, including sample identification numbers and borehole origins

Sample label	IODP sample ID	Hole-core-section-sample	Lava flow type	Sample label	IODP sample ID	Hole-core-section-sample	Lava flow origin
S1	CYL11155611	1571A-19-1-8	Top	S17	CYL11164511	1571A-35-1-80	Interior
S2	CYL11155631	1571A-19-1-89	Interior	S18	CYL11164521	1571A-35-2-53	Base
S3	CYL11156701	1571A-22-1-88	Interior	S19	CYL11164531	1571A-35-2-67	Base
S4	CYL11159231	1571A-24-1-27	Top	S20	CYL11165031	1571A-37-1-12	Top
S5	CYL11159241	1571A-24-1-51	Top	S21	CYL11165041	1571A-37-1-23	Top
S6	CYL11159251	1571A-24-1-75	Top	S22	CYL11165051	1571A-37-1-71	Interior
S7	CYL11159261	1571A-24-1-122	Interior	S23	CYL11182181	1572A-26-2-24	Interior
S8	CYL11160231	1571A-25-3-173	Top	S24	CYL11182191	1572A-26-3-126	Interior
S9	CYL11160241	1571A-25-3-106	Interior	S25	CYL11182201	1572A-25-4-21	Interior
S10	CYL11160251	1571A-25-3-139	Top	S26	CYL11182211	1572A-24-4-10	Interior
S11	CYL11160621	1571A-26-2-24	Top	S27	CYL11183201	1572A-27-3-24	Compound
S12	CYL11160631	1571A-26-2-37	Top	S28	CYL11190851	1572A-39-1-11	Interior
S13	CYL11162601	1571A-27-3-105	Interior	S29	CYL11190861	1572A-39-1-34	Interior
S14	CYL11163981	1571A-30-2-49	Interior	S30	CYL11191071	1572A-42-3-37	Top
S15	CYL11164481	1571A-34-2-48	Top	S31	CYL11191081	1572A-42-3-64	Top
S16	CYL11164501	1571A-35-1-137	Interior	S32	CYL11191091	1572A-44-4-31	Top

#### Shipboard analyses

Onboard sample characterisation using IODP Submethod D yielded a standard suite of physical property measurements (Planke et al. 2023c, 2023b), including the (wet and dry) density, derived grain density, and helium (He-)porosity data used in this study. For clarity, samples are referred to by their sample label throughout the remainder of the manuscript (e.g., CYL11159231 as sample S4; Table 1).

#### Thin sections and mineralogy

Polished, uncovered, and blue epoxy-impregnated thin sections were prepared for the 32 core plug offcuts by Independent Petrographic Services (IPC). The thin sections were analysed using an optical microscope and a Hitachi SU5000 FE-SEM Scanning Electron Microscope at the Goldschmidt Laboratory, Department of Geosciences, University of Oslo, to examine the mineralogy, textures and porosity in the plugs (Reed 2005). A Quantax XFlash 30 EDS Energy Dispersive Spectroscopy system was used to identify minerals and create elemental distribution maps.

#### Ambient gas permeability

Permeabilities were measured for 32 core plugs (Table 1). Ambient pressure single phase permeability was measured along the axial direction of core plugs using nitrogen (N_2_) gas as the pore fluid in a permeameter with a Hassler sleeve pressurized to 400 psi (~ 2.75 MPa) to avoid gas leakage around the sides of the core (e.g., Farrell et al. 2014). We refer to these measurements as ambient through the rest of the paper. Permeability measurements were Klinkenberg corrected (Klinkenberg 1941) to account for gas slippage.

### X-ray µCT scanning and multi-fluid flow experiments

#### S4 core plug analysis

Core plug S4 was chosen for further multi-fluid flow studies. Sample S4 represents one of the more altered yet permeable lava flow samples from the U1571 and U1572 boreholes, giving an opportunity to assess the impacts of alteration on fluid flow in basalt sequences. The core plug was recovered from the top of a basalt flow at a depth 165.17 m in Hole U1571A (Core 24R, Section 1) and is generally representative of the recovered flow top core plugs. The plug has a length of 4.0 cm, and a diameter of 2.52 cm. Density, volume and velocity measurements were repeated in preparation of the fluid substitution experiments, in addition to extended X-ray µCT scanning with multiple projection numbers and settings.

#### X-ray µCT scanning and computed physical properties

X-ray µCT scanning was done with a Metrology XT H 225 LC industrial scanner (Nikon) equipped with a 225 kV Ultrafocus 3C X-ray tube (3 μm spot size) and resulted in 1500–2000 projection per scan. Reconstruction of the projections into 3D volumes was done in Metrology X-TEK CT Agent XT (Nikon, v 3.1.11). Avizo 3D (v 2020.2 and v 2024.1) was used to further enhance and analyse the 3D volumes. Voltage-dependent shading corrections were updated daily with corresponding filters, i.e., 0.25 mm silver (Ag) filter. Comparison scans were taken at the start and end of each day to facilitate continuation. More advanced X-ray tomography analyses were performed through the PoresPy Python (Gostick et al. 2019) package and built-in Avizo 3D functions to derive computed porosity estimates.

#### Experimental set-up

**Fig. 3:**
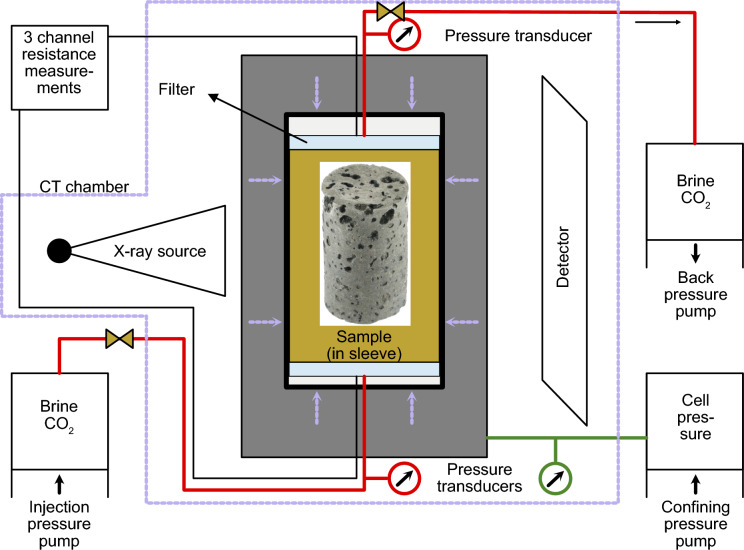
Schematic drawing of the set-up used during the X-ray µCT-integrated, multi-fluid flow experiments on sample S4

The experimental setup and procedure used in this study is based on the methods outlined in several previous publications (Alemu et al. 2013; Skurtveit et al. 2020, 2012). Core plug S4 was tested with an in-house-designed isotropic cell (Fig. 3; Norwegian Geotechnical Institute) to observe multi-fluid flow mechanisms under isotropic conditions (Alemu et al. 2013; Skurtveit et al. 2020, 2012). A Cold Shrink ^tm^ tube and nitrile rubber sleeve separated the core plug from the confinement oil and ensured fluid flow through the sample’s interior. Filters were placed on each end of the plug to prevent the migration of fines beyond the sample, with the flow in and out of the sample controlled by two Teledyne ISCO 260 D syringe pumps. A GDS pump controlled the confining pressure. Pressure sensors (Druck PDCR 4000 Series) at the inlet and outlet allowed the injection- and back-pressure to be continuously logged and monitored alongside the pump volumes. Resistance measurements were enabled by a National Instruments PXI series instrument that was connected to electrodes attached to the sample holders (platens) on either side of the sample and continuously measured alternating current with frequencies of 1000, 5000, and 10,000 Hz. The cell was placed inside a shielded X-ray µCT scanning cabinet, about ~ 20 °C in temperature, while the pumps remained outside. Small temperature variations in the cabinet occurred as the cabinet was closed during X-ray µCT scanning, potentially causing small density variations of the injected fluids. Resistance measurements were briefly stopped while scanning.

#### Experimental procedure

**Fig. 4:**
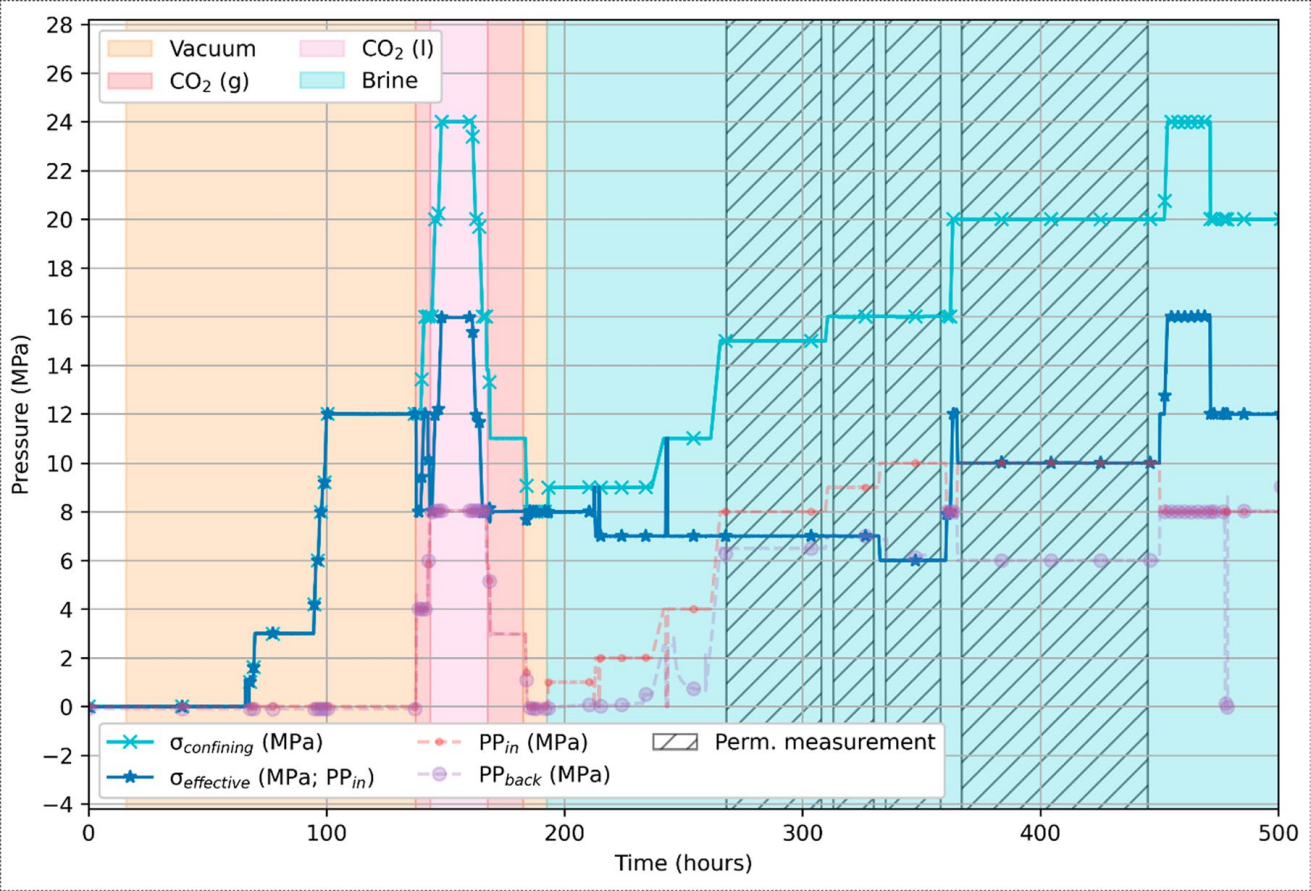
Overview of the pressure conditions for the vacuum (dry conditions), gaseous (g) and liquid (l) CO_2_, and brine phases of the fluid flow experiment on sample S4. PP in and back correspond to the injection and back pressure, respectively. The effective stress is calculated using the injection pressure (PP_in_). The hatched areas indicate the periods during which permeability to brine was measured

The test procedure involves four main phases: 1) a vacuum test phase for baseline measurements, 2) gaseous and 3) liquid CO_2_-saturated test phases, and 4) a brine-saturated phase. All four phases were conducted under varying pressure and stress conditions (Table 2, Fig. 4). The entire experiment lasted for 500 h (~ 20 days), of which approximately 300 h were used for the brine test phase and permeability-to-brine measurements. Incremental loading was applied to increase the average rate of loading while allowing time for the pore fluid to equilibrate. Stability of the sample and saturation were monitored through regular single- and dual-energy X-ray µCT scanning, as well as the resistance response.

**Table 2:** Pressure and stress conditions during the four testing phases of the multi-fluid flow experiments, as well as during the four brine permeability tests. Temperature was kept near-constant at room temperature

Test phase	Confining pressure (MPa)	Max back pressure (MPa)	Max injection pressure (MPa)	Effective pressure outlet (MPa)	Effective pressure inlet (MPa)	Max pore pressure difference (MPa)
Vacuum	0–12	0	0	0–12	0–12	0
CO_2_ (g)	8–13.8	5.8*	5.8*	8	8	~ 0
CO_2_ (l)	13.8–24	8	8	8	8	~ 0
Brine	8–24	8	10	8–16	8–16	4
Permeability test I	15	6.5	8	8.5	7	1.5
Permeability test II	16	7	9	9	7	2
Permeability test III	16	6	10	10	6	4
Permeability test IV	20	6	10	14	10	4

* Phase transition

The vacuum test phase included a loading/unloading cycle to set a baseline for the subsequent test phases in terms of resistance and X-ray µCT data, particularly to compare responses of the rock matrix as a function of pore and effective stress, as well as different fluids. Confining pressure was incrementally increased to 12 MPa effective stress, then reduced to 8 MPa in preparation for the CO_2_ test phase. During the latter, CO_2_ was introduced into the pore system through the inlet at 200 kPa, then gradually increased to 4 MPa. The confining pressure was then increased to 12 MPa, yielding an 8 MPa effective stress. Phase transition of CO_2_ from gaseous to liquid state was initiated by an incremental increase in pore pressure to 8 MPa while maintaining 12 MPa confining pressure. Phase transition throughout the sample took 2 h to complete, and time was allowed for the pore conditions to equilibrate. Subsequent loading/unloading led to a maximum 16 MPa effective stress in 4 MPa intervals. CO_2_ was then removed while confining pressure was simultaneously restored to 8 MPa. The liquid-to-gas phase transition took a similar amount of time as its reverse transition, as indicated by notable yet short delays observed in the response between the injection and backpressure sensors.

Following a full day of stable vacuum across the sample to ensure complete removal of CO_2_ from the pore space, the brine test phase was initiated using a 35 ppt NaCl solution. Brine was injected alternatingly from either end to increase the rate of saturation. An isotropic loading/unloading cycle was performed to study pore space and fluid flow changes. Absolute permeability to brine was measured through constant head permeability measurements for various effective pressure conditions and pore pressures, implementing Darcy’s law to calculate the permeability for the cylindric sample. Subsequent failure of the Cold Shrink sleeve was detected via X-ray µCT scanning during the final loading to 16 MPa effective stress/24 MPa confining pressure, which prevented additional permeability measurement during unloading and terminated the experiment.

## Results

### Bulk sample physical properties

The range in physical properties reported for the IODP U1571/U1572 sites (Planke et al. 2023b) and summarised in Table 3 highlights the heterogeneity between the lava flow types. The two lava flow base samples and 14 lava flow tops have considerably lower densities than the interiors and single compound flow sample. The mean He-porosity of the tested core plugs is 18.3%, ranging from 4.2 to 45.6%, based on onboard measurements (Planke et al. 2023b). Besides flow tops (~ 29%), the remaining lava flow types display generally low porosity (≤ 15%), following a similar inverse trend as observed in void ratios. Porosity appears inversely correlated to depth, with the shallowest samples (≤ 200 m bsf, mostly U1571) featuring the highest porosities. No such correlation is apparent for the interiors.

**Table 3:** Key physical property and (ambient) fluid flow parameters of the tested basalt samples

	n	Depth range (m bsf)^1,2^	Bulk density (g/cm^3^)^1^	Grain density (g/cm^3^)^1^	He Porosity (%)^1^	N_2_ (gas) Permeability (mD)	Void ratio^1^
Compound lava flow	1	231.46	2.627	2.871	13.2	0.0326	0.152
Lava flow base	2	220.5- 220.64	2.3445 (0.0559)	2.586 (0.077)	15.4 (0.566)	0.005050 (0.001061)	0.182 (0.00849)
Lava flow interior	14	146.29–287.04	2.702 (0.145)	2.896 (0.056)	10.1 (7.833)	0.0178 (0.353769)	0.112 (0.124)
Lava flow top	15	145.48–315.17	2.369 (0.193)	2.866 (0.092)	28.95 (8.958)	0.0251 (100.307)	0.4075 (0.181)
Sample S4	-	165.17	2.20	2.77	32.6	0.3672	0.484

Results are averaged by lava flow type, with the value range indicated by bracketed standard deviations

^1^Onboard IODP Submethod D measurements are available through LORE (Planke et al. 2023b). Note that densities are derived values from the moist-and-density method

^2^Based on IODP’s reported Core depth below Sea Floor A (CSF-A) metric

**Fig. 5:**
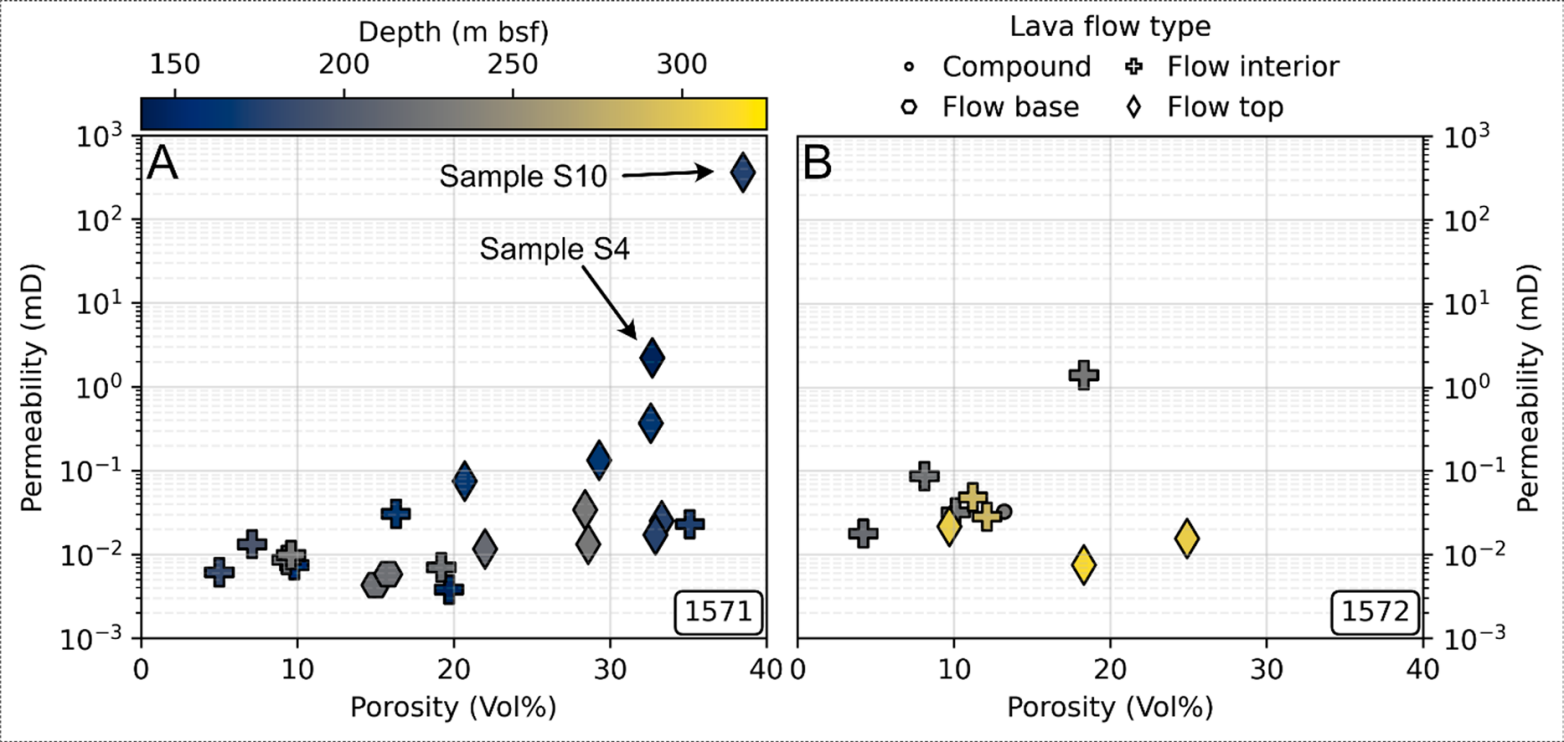
Measured fluid flow parameters for the 32 tested samples, plotted as permeability versus porosity for **A** Site U1571 and **B** Site U1572 samples. Shading indicates sampling depth; marker shape indicates lava flow type

In line with porosity, higher permeability is measured for lava flow tops than for interiors and bases (Fig. 5). Across both boreholes, ambient Klinkenberg-corrected gas permeabilities (N_2_) cover over 5 orders of magnitude and are mainly in the micro to millidarcy range (Table 3, Fig. 5). For Site U1571, permeability appears correlated with both porosity and lava flow type, albeit with significant scatter. Although a less clear trend is visible at Site 1572, lava flow tops feature high(er) permeability and porosity. At the high end of the permeability range, sample S10 (lava flow top) possesses a connected vesicle network resulting in both high porosity (38.5%) and gas permeability (361.9 mD), whilst a single highly vesicular sample (sample S8, 45.6%) gave no measurable pressure drop during testing, implying permeability > 10 Darcy. No depth-dependence is observed (Fig. 5).

**Fig. 6:**
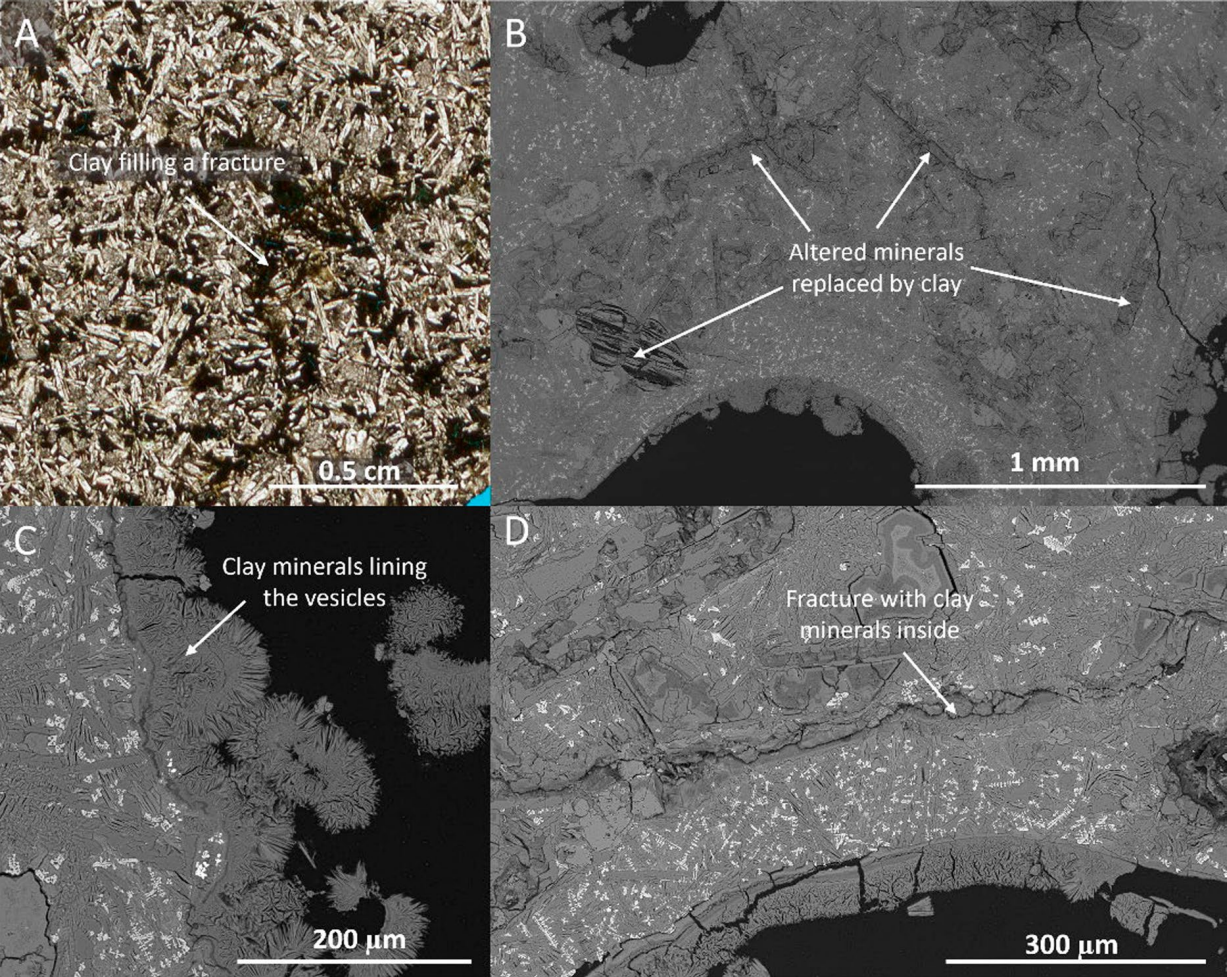
Microscope images indicating the presence of small-aperture features and their clay lining in samples S4 and S9. **A** Microscope image of clay filled fracture in sample S9 from borehole U1572. **B** Backscatter (BSE) image of sample S4 showing the alteration of minerals in the rock matrix. **C** Close-up of the clay minerals lining the vesicles in sample S4. **D** BSE image of small fracture in sample S4 that is at least partially filled with clay minerals

**Fig. 7:**
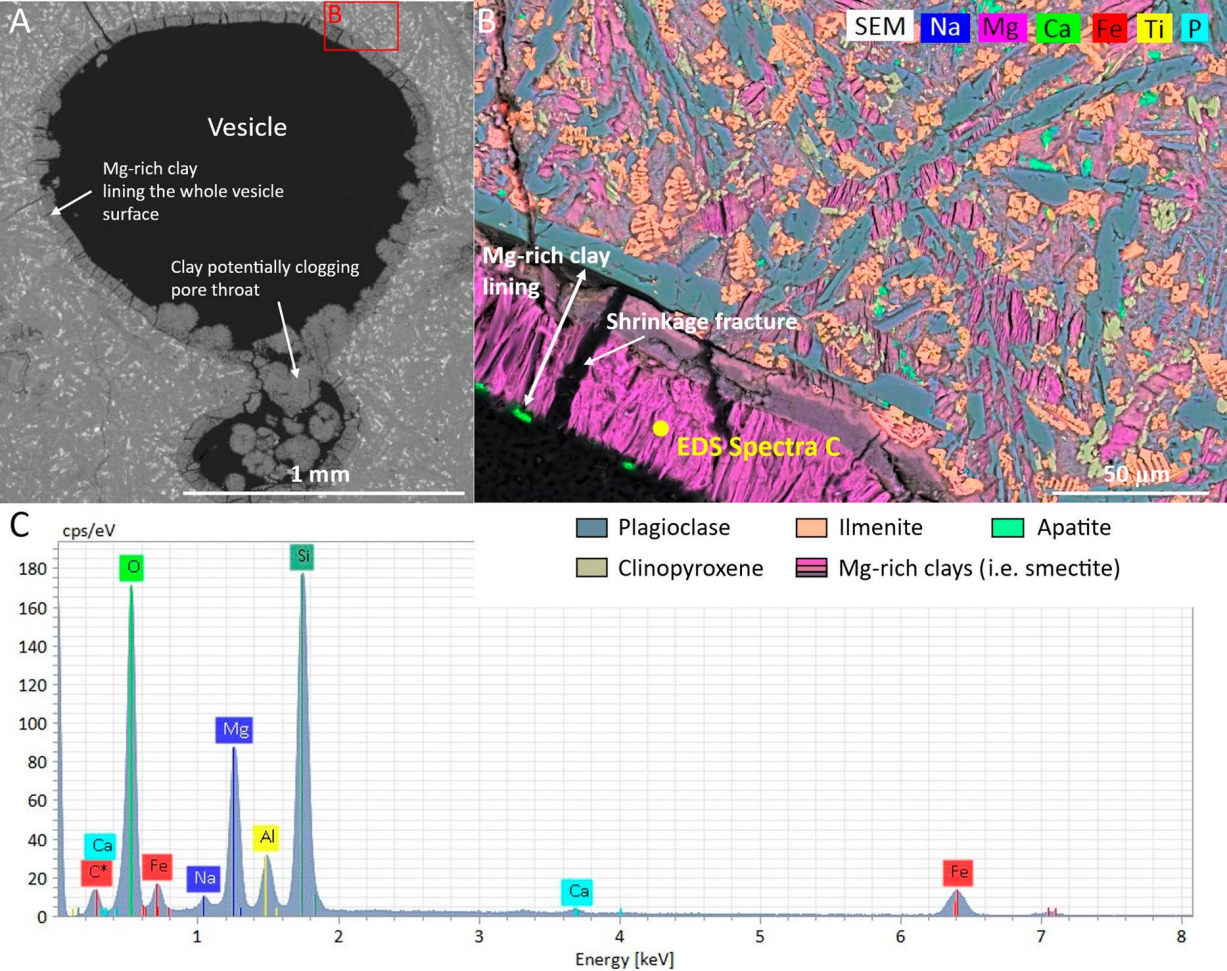
Pore-lining clay and elemental compositions in sample S4. **A** Backscatter (BSE) image of vesicle lined with clay minerals. The clay is potentially clogging the pore throat between the two pores. **B** Elemental map of marked area from image A showing the different minerals present in the sample, including Mg-rich clays (i.e., smectite), plagioclase, CPX-diopside, ilmenite, apatite, and Ca-carbonates. **C** EDS spectra of Mg-rich clay mineral lining the pore in image B

Petrographic analysis reveals that vesicle clay coatings are present in most plugs. Coatings usually range in thickness from around 30–100 µm (Fig. 7b, c), locally thicker, seemingly clogging pore throats and completely filling smaller vesicles (Fig. 7a). Locally, microfractures are filled with clays highlighting pervasive alteration (Fig. 6b–d). Notably, the clay layers seem to have a fibrous texture with fractures through it, radiating out, normal to the vesicle wall (Fig. 7b, d).

### Physical and mineralogical properties of sample S4

Onboard physical property and moist-and-density measurements on sample S4 found a He-porosity of 32.6%, and dry density and average grain densities of 1.87 and 2.77 g/cm^3^, respectively (Table 3), which are in line with subsequent laboratory validation prior to fluid flow testing.

**Fig. 8:**
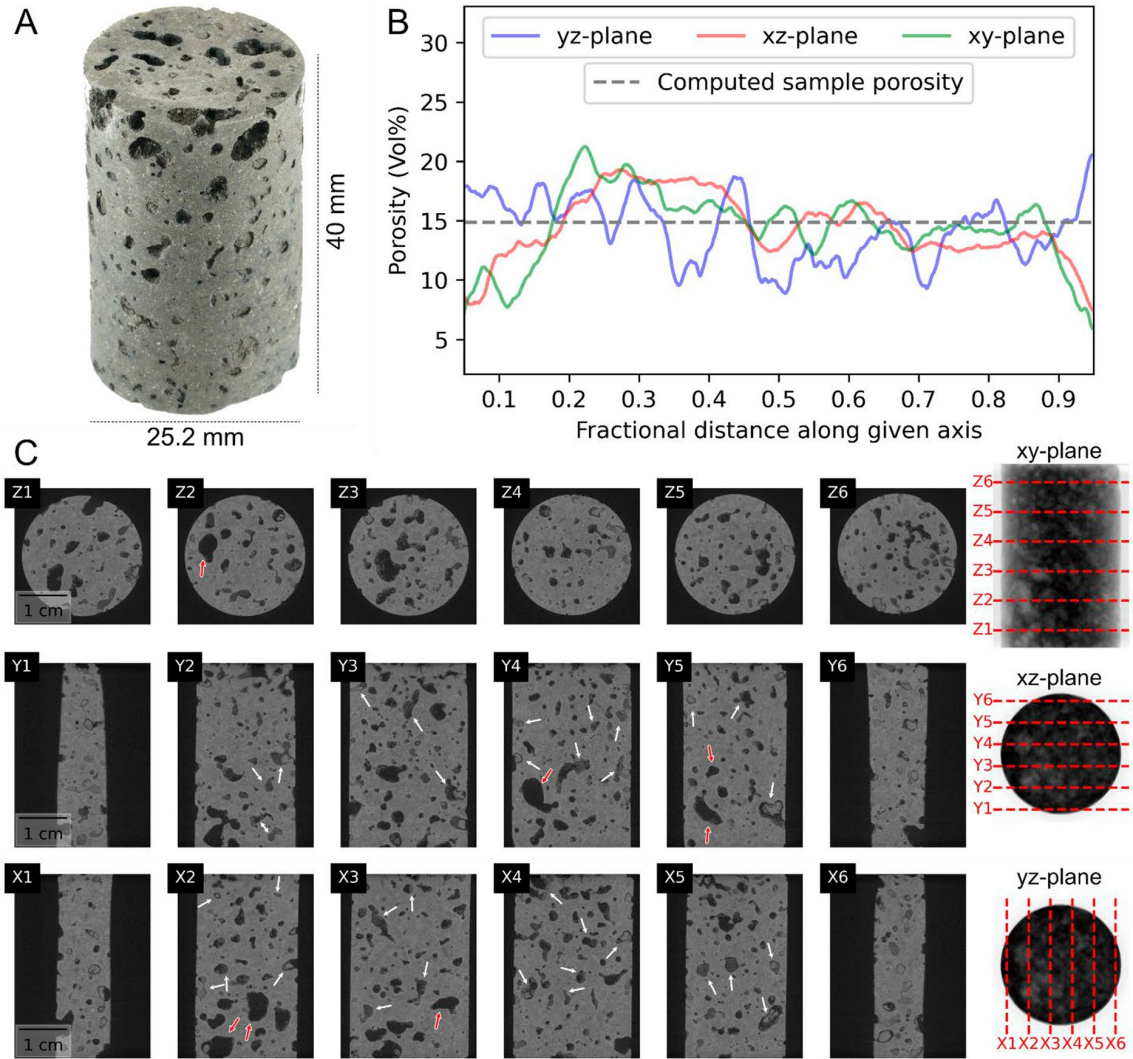
Physical properties of core plug S4. **A** Close-up photograph of sample S4. **B** Calculated 2D porosity as a function of the fractional distance along a given plane, as well as computed porosity for the whole volume (3D). **C** X-ray µCT imaging analysis and interval cross-section slices of core plug S4. White arrows highlight some of the clay-filled vesicles, identified as gray-scale infill of varying intensity, while red arrows indicate examples with only partial infill and/or surface lining

Petrographic analysis reveals that the lava flow top sample S4 is highly altered. The sample has a porphyritic texture with plagioclase and clinopyroxene glomerocrysts, and its groundmass crystals are fine-grained consisting of mainly plagioclase and clinopyroxene with some ilmenite crystals and minor amounts of apatite (Fig. 7). Mg-rich clays (likely smectite, Fig. 7c) are present as an alteration product in the groundmass from alteration of the silicate minerals and as partial to complete vesicle fills (Fig. 7a, b). As also noted for the other samples, clay coatings and fillings are prevalent in sample S4’s pore space. This is also evident from the pre-test X-ray µCT cross-section images shown in Fig. 8 where most, if not all, vesicles shown in the cross-section are either filled or coated with clays.

The X-ray µCT 3D volume analyses reveal generally little anisotropy in terms of porosity between the sample’s xy, yz and xz planes (Fig. 8b). The frequency distribution of pore and pore throat diameter show similar trends, and most pores and pore throats have a diameter below 5 mm. Only parts of the visible pores appear to be directly connected as only few of the micro-scale features (~ µm and smaller) are fully resolved. This is a known consequence of the micro-scale imaging resolution limit of the instrument (e.g., Botha and Sheppard 2016). Similarly, the sample’s computed average porosity of 14.9% is significantly lower than the measured 32.6% He-porosity (Table 3). In-depth analysis of the extracted pore space reveals most vesicles and pores to be isolated. The extracted pore space has a mean shape factor (Avizo calculation: shape_VA3d, A^3^/(36πV^2^)) of 12.56, which is defined as 1 for a perfect sphere (Fell et al. 2023). Complete exclusion of the clay lining/fillings from the matrix is difficult based on gray-scale intensity values alone, which may partly contribute to the observed discrepancy between the two porosity methods.

### Fluid substitution and flow testing

**Fig. 9:**
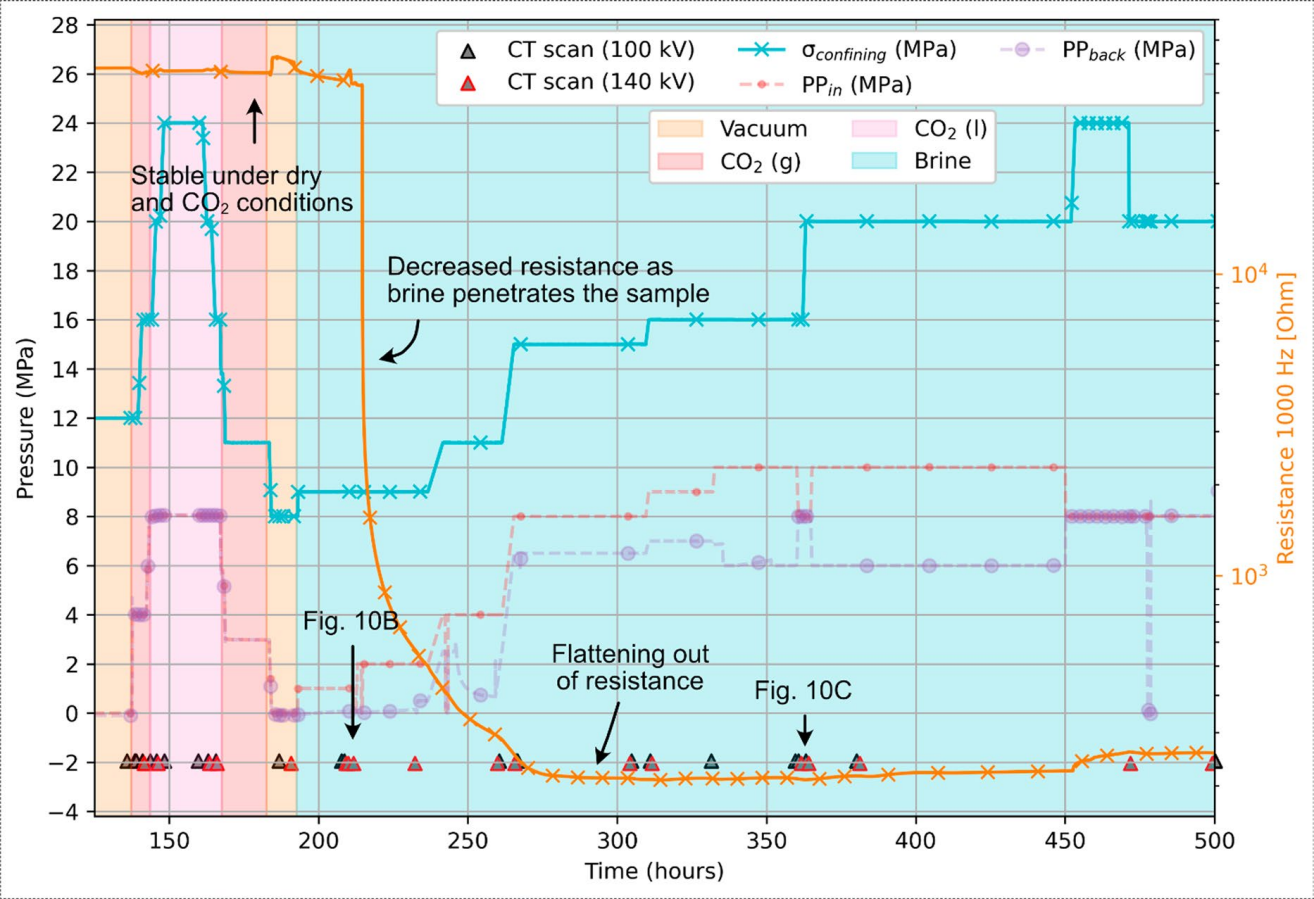
Resistance measurements and X-ray µCT scanning intervals during flooding with gaseous (g) and liquid (l) CO_2_ and brine. Confining, injection (PP_in_), and back (PP_back_) pressures are indicated as a reference. Acquisition times for the differential X-ray µCT scans shown in Fig. 10 are indicated

**Fig. 10:**
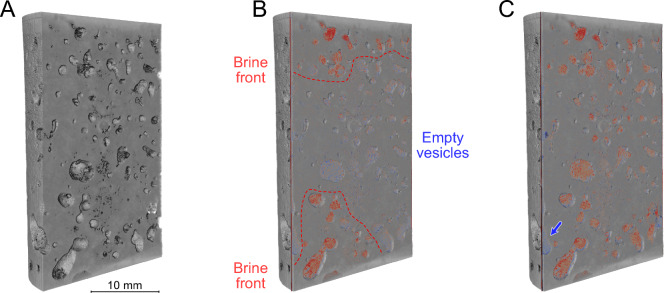
Differential X-ray µCT images of sample S4 **A** prior to, **B** during, and **C** after the brine saturation stage. The brine front on either end of the core plug is indicated by a red dashed line in (**B**), with red infill indicating an increase in measured density versus the vacuum reference (**A**). Note the blue arrow in (**C**) that highlights an isolated vesicle that remains empty even during full saturation of the sample. Image acquisition timings are indicated in Fig. 9

During fluid substitution and permeability experiments (Fig. 9), sample S4 pore space was exposed to the following conditions in sequence: 1) dry (i.e., vacuum, t = 15 h), 2) gaseous and liquid phase CO_2_ (t = 137 h), and 3) injection of brine (t = 191 h). Throughout, pump responses on either end of the sample remained stable. A delay in pore pressure response across the sample was only seen during phase transitions of CO_2_, (P = ~ 5.8 MPa) and the brine saturation phase. Pore pressure response otherwise remained instant across the sample. There was no obvious response in electrical resistance prior to injection of brine, when resistance (1000 Hz) dropped from ~ 50 kΩ to ~ 200 Ω during saturation (t = 80 h). X-ray µCT scanning confirmed the saturation of the sample yet also indicated the presence of a few disconnected vesicles. This is highlighted by the fluid front and empty vesicles in Fig. 10, underlining the presence preferential fluid flow pathways.

**Fig. 11:**
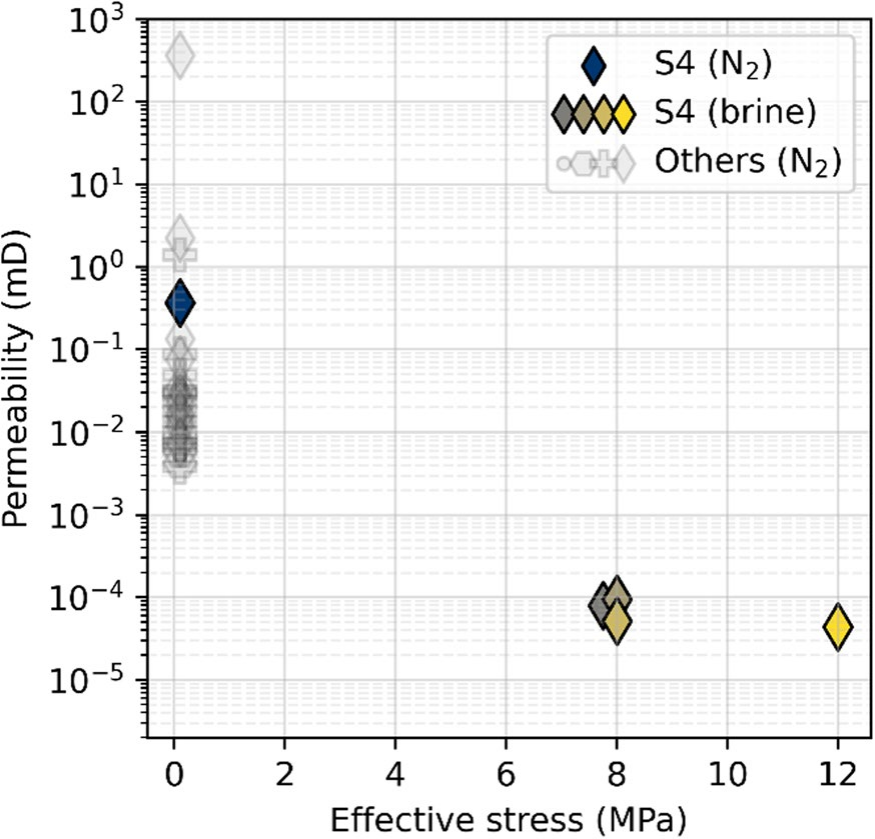
Permeability data for tested Skoll High basalt lava flow sample versus effective stress for each of the samples, including the four brine permeability measurements for sample S4 (each shaded differently)

Brine permeability measurements reveal sub-microdarcy levels of permeability (~ 10^–5^ mD; Table 4), roughly four orders of magnitude lower than the ambient gas permeability measurement on the same sample (Table 3). There appears little correlation between brine permeability and effective stress (Fig. 11), nor with confining stress (Table 4) at the stress levels employed during testing. Instead, the most significant change arises between the gas-measured permeabilities (at near-zero effective stress) and the four brine permeabilities (measured at higher stresses).

**Table 4:** Brine permeability results and pressure conditions during permeability testing of sample S4

Confining pressure	Injection pressure (MPa)	Back pressure (MPa)	Pressure difference (MPa)	Volumetric flow rate (m^3^/s, mean)	Permeability (mD)^1^	Permeability (m^2^)^1^
15	8.00	6.50	1.50	1.34E-12	7.93E-05	7.83E-20
16	9.00	7.00	2.00	2.12E-12	9.40E-05	9.28E-20
16	10.00	6.00	4.00	2.33E-12	5.18E-05	5.11E-20
20	10.00	6.00	4.00	1.97E-12	4.37E-05	4.31E-20

^1^Darcy’s law for a cylinder with an assumed brine fluid viscosity of 1.09E-3 Pa s, and length and diameter of the sample as defined in the text

## Discussion

The experimental work presented in this paper highlights a several orders of magnitude difference in permeability calculated from ambient tests versus that from a more comprehensive fluid substitution test with pressure confinement (Fig. 11). The results provide important input for discussing (1) the mechanisms involved in the observed core-scale fluid flow and permeability, specifically, the influence of stress conditions and fluid-rock interactions; and (2) upscaling and the implications of our findings for the Skoll High’s reservoir potential.

### Mechanisms governing flow at the core scale

The porosity–permeability relationships of the tested basalt plugs reveal that vesicle abundance alone is not a reliable predictor of fluid flow capacity, even when porosity exceeds the typical percolation threshold that enables fluid flow through ideal samples. This mismatch indicates that vesicle geometry and the size of connecting pore throats exert a stronger control on flow than total vesicular volume. For example, samples S8 (45.6%, > 10 Darcy) and S10 possess increased porosities and show measurable permeability increases, but most other plugs fall well short of such connectivity despite comparable porosity values. Indeed, only few of the samples’ vesicles and pores fulfil the percolation theory criteria of equal-sized, randomly distributed spherical pores (Bai et al. 2010; Mueller et al. 2005; Saar and Manga 1999). The lack of percolation amongst the tested samples may help explain why the general (gas) permeability of the altered lava flow samples is substantially lower than would be expected based on wireline and onboard fluid flow observations (IODP Exp 396—Porous basalt, 2021; Planke et al. 2023d).

The measured physical properties clearly vary between the distinct lava flow intra-facies (Table 3), reflecting their contrasting pore structures and subsequent alteration histories. Flow tops and bases typically display high primary vesicularity formed by bubble nucleation and migration during degassing and cooling at the flow margins and thus are more porous. In agreement, the tested interiors are less porous likely due to buoyancy driven bubble migration upwards into the flow top zones. As also evident from the wireline-derived porosity (Fig. 2), these primary differences in texture exert a first-order control on total porosity, yet they alone cannot explain the wide range of measured permeabilities.

Regardless of facies, pore throats are often found to be tortuous, kinked, or narrowed, preventing efficient networking even in otherwise vesicle-rich samples. The large discrepancy between helium porosity and porosity computed from X-ray µCT (Fig. 8) supports the interpretation that a significant fraction of pore space exists at the micro- to nanoscale, below the imaging resolution, and may be effectively isolated from fluid flow. Such differences between methods are not uncommon (Haijun et al. 2023; Nara et al. 2011; Yamamoto et al. 2024), and they highlight that connectivity is primarily facilitated by micro-scale elements. It is at these scales that mechanical stresses, e.g., through fracture closure, and physicochemical processes, including fines-migration, water adsorption, and associated swelling, are especially potent (Callow et al. 2018; Colombier et al. 2017; McGrail et al. 2017; Millett et al. 2024; Raza et al. 2022; Schaef et al. 2011). Even minor changes in stress or fluid composition may close or block key pore throats and flow corridors (Davudov and Moghanloo 2018; Skurtveit et al. 2012), abruptly halting flow and leading to multi-order magnitude changes in permeability.

Pressure-dependent permeability evolution is commonly linked to the closure of fractures at both macro- and micro-scales (e.g., Fortin et al. 2011; Nara et al. 2011; Stanton-Yonge et al. 2023). In basalts, macro-fractures are typically more prone to closure at low effective pressures (< tens of MPa), while micro-fracture closure dominates at higher stresses (Nara et al. 2011). Given the relatively low-pressure regimes employed in our experiments, macro-fracture closure would be expected to dominate. However, such features are absent in the tested plug (Fig. 8), and basalts generally show limited stress sensitivity due to their high strength (Fortin et al. 2011; Heap et al. 2018; Nara et al. 2011). Crucially, analysis of the CO_2_ and brine flow-through data indicates only a minor adverse permeability response to increasing confining pressure, effective stress, or pore pressure gradient (Fig. 11**, **Table 4). Thus, mechanical effects alone are insufficient to explain the observed four-order magnitude permeability decrease.

Alteration products, particularly swelling clays, are known to exert a first-order control on permeability and fluid flow. Smectite and related clays are frequently observed in older and more altered basalts, such as those tested here, where they line vesicles and microfractures, narrowing pore throats and restricting effective connectivity. Their behaviour is strongly dependent on fluid composition and stress state, and swelling, remobilization, and fines migration may all act to severely reduce permeability (Aksu et al. 2015; Callow et al. 2018; Foster 1954; Franzson et al. 2008; Norrish 1954; Suárez et al. 2023; Zhang et al. 2018; Zhou et al. 1996). In addition to the observed swelling, fines migration was also observed during comparisons of X-ray CT images taken at different stages during brine injection. Thus, the prevalence of vesicle- and fracture-lining clays in the Skoll High samples (Fig. 6, 7, 8, and 10) and subsequent rock-fluid interactions therefore provides a plausible explanation for the multi-order discrepancy observed between gas and brine permeabilities.

Across-sample pressure responses during fluid substitution testing of sample S4 corroborate this further. Prior to introducing brine, across-sample pressure responses remained constant during injection of both gaseous and liquid CO_2_, even at higher stresses, indicating no major obstruction of flow (Fig. 9**)**. The response lagged only briefly during the two CO_2_ phase transition phases, restoring directly after. CO_2_ sorption by clays may have played a role for the delayed pore response herein, characterised by a staggered transition and may be due to very localised and temporary clogging of the sample, by a similar process as described by (Busch et al. 2016, 2008; Heap et al. 2018). In contrast, a sharp decrease in permeability was immediately observed upon initial brine injection, as reflected by delayed saturation and reduced flow-through rates at low effective stress (Fig. 9, 11). Flow rates remained substantially reduced thereafter. A residual CO_2_ entrapment effect is unlikely, given that samples were held under vacuum for at least 24 h after CO_2_ flushing to ensure its removal. We therefore interpret the discrepancy between gas and brine permeabilities to reflect (brine-induced) fluid-rock interactions, specifically narrowing and closure of pore throats by fines migration and swelling within clay-lined vesicles and fractures. Similar processes in basalts have been described by others (e.g., Aksu et al. 2015; Callow et al. 2018; de Jong et al. 2014; Heap et al. 2018), but seldom at the magnitude observed here.

Methodological constraints must be acknowledged. Given constraints in the experimental setup, no permeability quantification was done during injection of CO_2_. Further, two of the four brine flow tests were conducted at pore pressure differences (ΔPf) slightly exceeding the recommended threshold of ΔPf < 1/3 Pf mean for ensuring laminar flow conditions (Bernabe et al. 1982; Brace et al. 1968). This raises the possibility of minor non-laminar effects during these measurements. However, even if present, such effects are unlikely to explain the observed four-order magnitude difference between gas and brine permeabilities. Instead, the abrupt change in flow-through points to intrinsic flow control at the pore scale, where micro-scale features and clay sealing thereof dominate fluid flow capacity.

Indeed, laboratory testing of samples where clay is involved is notoriously difficult, especially in terms of upscaling and applicability of findings under in situ conditions. Swelling and remobilisation in the Skoll High core plugs is likely further exacerbated by two sample recovery and handling protocols. Firstly, fluid-rock interactions are heavily influenced by in situ pore water chemistry, which is often difficult to ascertain and can be regionally and even locally variable. As no clear constraints were present for the tested interval, a seawater-like fluid was used during brine testing, which may have triggered swelling by the changing ionic conditions (Callow et al. 2018), resulting in the closure of the sub-micro pore space. Secondly, as is typically the case, core plugs were to a certain extent dried prior to testing, as is also evident from the outwards radiating fractures as seen in Fig. 7**.** Subsequent rehydration of dehydrated clays during brine injection is likely to have induced additional swelling and fines migration. This may explain the observed differences in CO_2_ and brine flow rates between their respective injection phases.

The observed discrepancy is not unique to the Skoll High basalts and reflects a broader methodological limitation that affects the interpretation of basalt reservoirs worldwide. Namely, that the use of inert gas permeability testing of air- or oven-dried samples does not adequately capture the behaviour of swelling clays or fines migration, as the measured permeability may be overestimated by up to several orders of magnitude (this study; Callow et al. 2018; Heap et al. 2018). Specifically, the results urge precaution when assessing the CO_2_ storage capacity and injectivity potential of possible storage sites where significant alteration is present. This includes those targeting volcanic reservoirs in the Deccan Traps (Liu et al. 2022), the North Atlantic Igneous Province (Rosenqvist et al. 2024, 2023), the Paraná Etendeka Igneous Province (Guimarães Titon et al. 2021), along with any other scenario where swelling minerals such as smectite may be common.

### Upscaling, macro-scale elements and reservoir implications

Taken together, our results demonstrate that the observed plug-scale permeability variations are primarily governed by pore-scale geometry and the physicochemical behaviour of swelling clays, with only limited sensitivity to stress conditions. However, while these micro-scale processes dominate the measured matrix permeability, they represent only one component of a much more complex hydraulic system. To evaluate how such intrinsic controls manifest under in-situ conditions, and how they interact with larger-scale structural features and natural alteration, it is necessary to consider the upscaling of the observed processes to the flow-unit and reservoir scale.

Swelling of clays hosted within the recovered volcanic lithologies, including lava flow tops, interiors, and bases, was noted onboard during core handling and characterisation, providing some insights to the possible in-situ swelling behaviour. The most apparent swelling occurred almost immediately after contact of cut core surfaces with water, suggesting a chemical and/or stress dependence in line with previous observations in similar settings elsewhere (e.g., Madsen and Müller-Vonmoos 1989; Sridharan et al. 1986; Wilson et al. 2014). These observations imply that the clays are largely unswollen or only partially swollen under undisturbed subsurface conditions, with swelling and associated clogging, likely initiated by external triggers such as pore-pressure and temperature fluctuations or chemical disequilibrium during injection (Busch et al. 2016; Tangparitkul et al. 2020). Of particular relevance is the co-injection of high-salinity seawater, a proposed cost-effective approach for offshore CO_2_ storage, which may disturb the equilibrium of smectite-rich assemblages. Osmotic swelling, ion exchange, and fines mobilisation could further reduce pore-throat diameters and effectively close microfractures, thereby amplifying the permeability decline observed by us and others (e.g., Callow et al. 2018). Conversely, thermal or stress perturbations may induce micro-fracturing and transient increases in permeability. The interplay between these opposing processes defines the transient permeability of the basalt pile and ultimately its capacity for sustained injection. Importantly, there is ample evidence elsewhere of fluid flow through micro- and nanopores of altered vesicular basalts, even in sequences as tight as those studied here. Oil and/or carbonate filled vesicles are documented, for example, in the field near Nuussuaq, West Greenland (Christiansen 2011; Christiansen et al. 2020) and from offshore core data offshore Brazil (Badejo and Linguado fields, Marins et al. 2022).

Following the observation of post-recovery swelling coupled with the very low matrix permeability of the drill core plugs, it can be inferred that an effective seal is present that separates the targeted basaltic intervals from overlying sequences. Otherwise, swelling would likely have occurred already, given the shallow depth, proximity to the seafloor, and probable seawater incursion in the recent geological past. However, both borehole and drill core observations suggest potentially good vertical and lateral connectivity across the sequences. Vertical fluid connectivity is evident from strontium isotope analysis across the U1571A borehole, covering both vesicular and massive (i.e. interior) basalt sequences (Polteau et al. in press). Past fluid flow is also evident from carbonate clay coatings (Fig. 6) and core-section fracture network infills (Planke et al. 2023d), suggesting connectivity and even natural geological carbon sequestration at some point in the geological past.

Such observations, as also made at ODP Hole 642E, reveal that macro-structural elements can locally override the low matrix permeabilities and shift the focus of further appraisal towards understanding transient permeability and macro-scale flow corridors. Both borehole descriptions and core observations from Sites U1571 and U1572 document coalesced vesicular networks and natural fractures that extend across lava flow interiors and margins (Planke et al. 2023b). These larger-scale features provide tortuous yet effective fluid pathways that contrast sharply with the tight behaviour measured in most core plugs, especially when (inter)connected over larger intervals (Fornero et al. 2023; Lamur et al. 2017; Millett et al. 2024; Pollyea et al. 2014).

Given their similarity, findings from the successful Wallula pilot project may offer useful insights to the Skoll High’s sequestration potential and bridge the ODP Hole 642E and U1571A isotope observations with those presented here. From X-ray CT scans of side-wall cores from the Wallula injection site, it is clear that the vesicles are coated with some pre-existing precipitates. Although no composition is specified, they appear very similar to the clay coating observed on the Skoll High samples (Fig. 7) (McGrail et al. 2017). Post-injection sampling, analyses and modelling following a 25-day injection period in 2013 show that approximately 60% of the scCO_2_ was mineralized in two years, and that the scCO_2_ entered and mineralized in vesicles (Kelly et al. 2024; Shen et al. 2023; White et al. 2020), underlining that sequestration potential persists even where clays coatings may be prevalent. While the injectivity of the 20 m-thick, flow-top-dominated reservoir target (~ 75–150 millidarcies) resembles that of the flow estimate for the more permeable sections of ODP Hole 642E, the surrounding basalt flow facies at both Wallula and Skoll High (this study) are generally low (~ microdarcies), providing an important secondary trapping mechanism to any future sequestration (McGrail et al. 2009). Thus, as also shown for the wider Colombia River basalts (Jayne and Pollyea 2018), the prevalence and potential of fracture permeability is key to mass, fluid and heat flow across the Vøring Margin, underlining the need to understand fracturing, fracture (flow) processes and transient permeability in the Skoll High lava pile to fully appraise the margin’s sequestration potential.

## Conclusion

In this contribution we present laboratory petrophysical testing and fluid flow observations of 32 core plugs retrieved from vesicular basaltic lava flows on the Skoll High, Vøring Margin during IODP Expedition 396 and conclude that:

1. Only six of the 32 tested samples feature porosities above 30%, i.e., the percolation threshold at which high connectivity of vesicle networks typically occurs.
2. The matrix permeability (to gas N_2_) of the tested Skoll High basaltic lava flows at the mini-core scale is dominantly low but highly variable in the low micro to millidarcies range (0.0038–361.9 mD). Higher permeability is measured for flow tops than for interiors and bases.
3. The vesicular pore networks of the samples appear to be largely unconnected, as also evident from X-ray µCT imaging and SEM imaging. Micro-scale elements seem to play an outsized role in determining connectivity between the vesicles, and, ultimately, govern effective permeability.
4. Brine permeability was found to be four orders of magnitude lower than the gas permeability (N_2_), questioning the appropriateness of this commonly applied method and applicability of legacy data as input to reservoir characterisation on the Mid-Norwegian Continental Margin and elsewhere. There is a need to appraise hydraulic properties under in situ-like (and wet) conditions to avoid the overestimation of capacity and injectivity potential, both crucial for effective carbon sequestration site appraisal.
5. The low matrix permeability of the tested plugs, their high overall degree of alteration, and the prevalence of swelling clays negatively affects the sequestration potential of the Skoll High and similar altered basaltic sequences in the North Atlantic. However, the scaling of the test plugs relative to the often-complex multi-scale pore networks, and the impacts of both primary cooling joints and secondary tectonic fractures require detailed appraisal before a fully comprehensive assessment can be made.

## Data Availability

All shipboard data are available from the IODP LIMS Online Reports Portal (LORE; (https://web.iodp.tamu.edu/LORE/)). Additional data will be made available upon request.
